# Local Application for the Relief of Erysipelas

**Published:** 1881-07

**Authors:** 


Local Application for the Relief of Erysipelas.—

| | |
|---|---|
| R.—Acid Carbolic, - - - | 1 part |
| Spt. Vini, - - - - | 1 " |
| Olei Terebinth, - - - | 2 " |
| Tinct. Iodine, - - - - | 1 " |
| Glycerine, - - - - | 5 " |

M.—Sig. Paint on the surface and cover the painted part with a thin layer of fine carded cotton batting. The applications are painless, and the good effects can be seen on the second or third day.—*Pittsburg Medical Journal.*

